# Comparison of the NG-Test Carba 5, Colloidal Gold Immunoassay (CGI) Test, and Xpert Carba-R for the Rapid Detection of Carbapenemases in Carbapenemase-Producing Organisms

**DOI:** 10.3390/antibiotics12020300

**Published:** 2023-02-02

**Authors:** Danxia Gu, Zelin Yan, Chang Cai, Jiaping Li, Yanyan Zhang, Yuchen Wu, Jiaxing Yang, Yonglu Huang, Rong Zhang, Yongning Wu

**Affiliations:** 1 Laboratory Medicine Center, Department of Clinical Laboratory, Zhejiang Provincial People’s Hospital, Affiliated People’s Hospital, Hangzhou Medical College, Hangzhou 310014, China; 2 Department of Clinical Laboratory, Second Affiliated Hospital of Zhejiang University, School of Medicine, Hangzhou 310009, China; 3 College of Animal Science and Technology, College of Veterinary Medicine, Zhejiang Agricultural and Forestry University, Hangzhou 311300, China; 4 NHC Key Laboratory of Food Safety Risk Assessment, China National Center for Food Safety Risk Assessment, Research Unit of Food Safety, Chinese Academy of Medical Sciences (2019RU014), Beijing 100022, China

**Keywords:** KPC variants, carbapenemase, rapid detection, NG-test Carba 5, Xpert Carba-R, evaluation

## Abstract

Carbapenem-resistant Enterobacterales (CRE) are increasingly recognized as an urgent public health concern. The rapid and accurate identification of carbapenemases could provide insights into antimicrobial therapy and infection control. In this study, we evaluated the efficacy of three different methods, including the NG-test Carba 5, colloidal gold immunoassay (CGI) test, and Xpert Carba-R assay, for the rapid detection of five carbapenemases (KPC, NDM, IMP, OXA-48, and VIM). A total of 207 Gram-negative strains collected from patients and hospital sewages were tested. The presence or absence of carbapenemase genes in the whole-genome sequences was used as the gold standard for evaluating the accuracy of the above-mentioned three methods. Among the 192 strains carrying only one carbapenemase gene, the accuracies of the NG-Test Carba 5, CGI test, and Xpert Carba-R were 96.88% (95% CI, 93.01–98.72%), 96.88% (95% CI, 93.01–98.72%), and 97.92% (95% CI, 94.41–99.33%), respectively. Xpert Carba-R was able to detect all 13 types of KPC variants, including KPC-2, KPC-3, KPC-25, KPC-33, KPC-35, KPC-51, KPC-52, KPC-71, KPC-76, KPC-77, KPC-78, KPC-93, and KPC-123, with a detection sensitivity of 100.00% (95% CI, 96.50–100.00%), a specificity of 100.00% (95% CI, 92.38–100.00%), and a κ index of 1.00. For IMP, Carba 5 was superior to the other two methods, with a sensitivity of 100% (95% CI, 71.66–100.00%), a specificity of 100% (95% CI, 97.38–100.00%), and a κ index of 1.00. For the remaining 15 strains carrying two or three kinds of carbapenemase genes, Carba 5 performed the best, which accurately identified all the target genes, followed by Xpert Carba-R (12/15, 80.00%) and the CGI test (10/15, 66.67%). Therefore, all three assays demonstrated reliable performances in carbapenemase detection, and Xpert Carba-R should be recommended for the detection of KPC variants, especially for patients at a high risk of infections caused by ceftazidime/avibactam-resistant strains. IMPORTANCE: CRE was listed as one of the top three pathogens that are in critical need of new antibiotics by the WHO. The rapid and accurate identification of carbapenemases is important for antimicrobial therapy and infection control. In recent years, new beta-lactam/beta-lactamase inhibitor combinations such as ceftazidime/avibactam (CZA) have been approved by the Food and Drug Administration (FDA) to cope with CRE challenges. CZA was effective against class A, class C, and some class D enzymes such as OXA-48-like. However, CZA-resistant KPC variants emerged at an alarming speed, which posed a new challenge for the accurate identification of KPC variants. In this study, we evaluated the performance of two lateral flow immunochromatographic assays, namely, NG-test Carba 5 and the CGI test, and the automated real-time quantitative PCR Xpert Carba-R in the rapid detection of carbapenemases. Notably, 13 types of KPC variants were enrolled in this study, which covered most KPC variants discovered in China. Carba-R was superior to NG-teat Carba 5 and the CGI test; it was able to detect all of the included KPC variants, including KPC-2, KPC-3, KPC-25, KPC-33, KPC-35, KPC-51, KPC-52, KPC-71, KPC-76, KPC-77, KPC-78, KPC-93, and KPC-123.

## 1. Introduction

Antimicrobial resistance is one of the leading public health threats of the 21st century. According to a recent report [1], an estimated 4.95 million (3.62–6.57) deaths were associated with bacterial antimicrobial resistance in 2019, among which 1.27 million (95% UI, 0.911–1.71) were attributed to bacterial antimicrobial resistance. Carbapenem-resistant Enterobacterales (CRE) which undergo global transmission are increasingly recognized as an urgent threat. Data from the China CRE Network in 2015 indicated that the incidence of CRE infection was 4.0/10,000, and the mortality was 33.50% [2]. In the USA, CRE admission was 57 per 100,000 admissions, and the 30-day mortality was 24% [3]. According to the World Health Organization (WHO), CRE is one of the top three pathogens that are in critical need of new antibiotics.

The production of carbapenemases is the major cause of carbapenem resistance in CRE strains [4]. Carbapenemases typically belong to three classes of β-lactamases: classes A, B, and D. Among class A carbapenemases, *Klebsiella pneumoniae* carbapenemases (KPCs) are the most prevalent. NDM, IMP, and VIM are the representative class B metallo-β-lactamases. Oxacillinase (OXA)-48-like β-lactamases are the most common Class D carbapenemases. Han et al. reported that carbapenemases were detected in 97.43% of CRE strains in China from 2016 to 2018, with KPC-2 being dominant (51.55%), followed by NDM (35.72%) and OXA-48-like (7.27%) [5].

Infections caused by CRE strains are often difficult to treat. New beta-lactam/beta-lactamase inhibitor combinations such as ceftazidime/avibactam (CZA) have been approved by the Food and Drug Administration (FDA) to cope with CRE challenges. CZA can exert a potent inhibitory function against class A, class C, and some class D β-lactamases (such as OXA-48-like), but it is ineffective against class B metallo-β-lactamases [6]. The rapid and accurate identification of carbapenemases can provide insights into antimicrobial therapy and infection control.

The Clinical and Laboratory Standards Institute (CLSI) recommended several tests for the determination of carbapenemases in Enterobacterales and *Pseudomonas aeruginosa*. Carba NP is rapid and can be finished in two hours, but it requires some reagents which have to be prepared in the laboratory and have short shelf lives. Modified carbapenem inactivation methods (mCIM) and mCIM with eCIM are based on overnight cultivation. Various methods have been developed for the rapid detection of carbapenemases, such as the biochemical assay-based Nitro Speed-Carba NP Test [7], lateral flow immunochromatographic assay NG-test Carba 5 [8], and automated real-time quantitative PCR Xpert Carba-R [9]. In this study, the performances of the NG-test Carba 5, colloidal gold immunoassay (CGI) test, and Xpert Carba-R assay were analyzed with carbapenemase-producing strains isolated from patients and hospital sewages.

## 2. Results

Of the 207 strains tested in this study, 192 strains carried a single carbapenemase gene covering 19 types of carbapenemase-encoding genes, including *bla*_KPC_ (120 *bla*_KPC-2_ and 13 *bla*_KPC_ variants), *bla*_NDM_ (19 *bla*_NDM-1_, 24 *bla*_NDM-5_, and 1 *bla*_NDM-13_), *bla*_IMP_ (2 *bla*_IMP-1_, 4 *bla*_IMP-4_, 2 *bla*_IMP-8_, 1 *bla*_IMP-25_, 2 *bla*_IMP-26_, and 2 *bla*_IMP-30_), and *bla*_OXA-48_-like (1 *bla*_OXA-181_ and 1 *bla*_OXA-232_). Additionally, 15 strains co-producing two or three types of carbapenemases were also tested Supplementary Tables S1 and S2.

Regarding strains with single carbapenemase genes, the overall accuracy of the NG-Test Carba 5, CGI test, and Xpert Carba-R was 96.88% (95% CI, 93.01–98.72%), 96.88% (95% CI, 93.01–98.72%), and 97.92% (95% CI, 94.41–99.33%), respectively. The sensitivity and specificity varied with different types of carbapenemases, as shown in Table 1. For the detection of different variants of KPC, Xpert Carba-R was superior to the other two methods, with a sensitivity of 100.00% (95% CI, 96.50–100.00%) and a specificity of 100.00% (95% CI, 92.38–100.00%). Compared to the WGS results, the κ index of Xpert Carba-R was 1.00. NG-test Carba 5 missed six target Carbapenemases, while the CGI test missed two targets, leading to a sensitivity of 95.49% (95% CI, 90.02–98.15%) and 98.50% (95% CI, 94.12–99.74%), respectively. The κ indexes of NG-test Carba 5 and the CGI test were 0.93 and 0.98, respectively. As for NDM, the two immunochromatographic assays accurately detected all of the target carbapenemases with a sensitivity of 100.00% (95% CI, 90.00–100.00%) and a specificity of 100.00% (95% CI, 96.85–100.00%), whilst Xpert Carba-R missed two targets, with one false-negative result and one false-positive result, leading to a sensitivity of 97.73% (95% CI, 86.49–99.88%) and a specificity of 99.32% (95% CI, 95.73–99.96%). Compared with the results of the WGS, the κ indexes of the NG-test Carba 5, CGI test, and Xpert Carba-R were 1.00, 1.00, and 0.97, respectively. For IMP, NG-test Carba 5 performed the best; it identified all the variants with a sensitivity of 100% (95% CI, 71.66–100.00%) and a specificity of 100.00% (95% CI, 97.38–100.00%). This was followed by Xpert Carba-R, which missed three targets and exhibited a sensitivity of 76.92% (95% CI, 45.98–93.83%), and the CGI test, which missed four targets and had a sensitivity of 69.23% (95% CI, 38.89–89.65%) and a specificity of 100.00% (95% CI, 97.38–100.00%). The κ indexes of the Carba 5, CGI test, and Xpert Carba-R were 1.00, 0.81, and 0.86, respectively. For the OXA-48-like carbapenemase, all three methods reported the detection of OXA with a κ index of 1.00. As for VIM, only one *Enterobacter cloacae* strain co-producing VIM-1 and NDM-1 was included in the current study, which was accurately detected by all three methods.

Except for KPC-2 and KPC-3, 11 other types of KPC variants were included, as shown in Table 2, covering KPC-25, KPC-33, KPC-35, KPC-51, KPC-52, KPC-71, KPC-76, KPC-77, KPC-78, KPC-93, and KPC-123. NG-test Carba 5 detected 6 out of the 12 KPC variants, including KPC-3, KPC-25, KPC-35, KPC-51, KPC-78, and KPC-93. As for the CGI test, KPC-76 and KPC-123 were not detected. Xpert Carba-R accurately identified all 12 targets. Overall, KPC-3, KPC-25, KPC-35, KPC-51, KPC-78, and KPC-93 could be detected by all three methods.

For IMP, six types of IMP variants were included, covering IMP-1, IMP-4, IMP-8, IMP-25, IMP-26, and IMP-30 (shown in Table 3). NG-test Carba 5 detected all IMP variants, while the CGI test missed one IMP-8-producing *Aeromonas hydrophila*, one IMP-26-producing *K. penumoniae*, and two IMP-30-producing *K. pneumoniae*. Regarding Xpert Carba-R, one IMP-4-producing *A. hydrophila* was misidentified as NDM, and two IMP-8-producing strains (1 *A. hydrophila* and 1 *P. aeruginosa*) were not detected.

The detection results for strains with multiple carbapenemase genes are shown in Table 4. NG-test Carba 5 detected all 15 strains accurately. For the CGI test, three *Klebsiella* spp. strains co-producing KPC-2 and IMP-4 were reported as KPC-producing, and two *K. oxytoca* strains co-producing NDM-1 and IMP-4 were reported as NDM-producing. Regarding Xpert Carba-R, one *Klebsiella michiganensis* with the coexistence of KPC-2, NDM-1, and IMP-4 was detected as KPC and NDM. Additionally, two *P. aeruginosa* strains co-producing IMP-45 and NDM-1 were reported as IMP-producing.

## 3. Discussion

The rapid and accurate detection of carbapenemase is of significant importance to tackling the threat caused by the global transmission of multidrug-resistant bacteria. Carbapenemase detection is not only important for effective clinical chemotherapy against infections but also for the prevention and control of carbapenemase-producing strains in healthcare-associated environments. Several methods have been developed for the detection of carbapenemase. Some of the methods, such as the phenotypic characterization of the carbapenem-hydrolyzing activity, involve a complex operation with a long turn-around time, and their application in clinical laboratories is therefore restricted. In this study, the performances of three commercial kits for the detection of carbapenemases (KPC, NDM, IMP, VIM, and OXA-48-like), namely, the NG-test Carba 5, CGI test, and Xpert Carba-R, were evaluated with clinical and hospital sewage carbapenemase-producing strains.

As reported, all three assays were able to shorten the turnaround time to less than two hours and directly identify carbapenemases from clinical samples [8,9,10,11,12,13]. Overall, the three methods demonstrated an excellent detection performance among CRE strains. With regard to *Klebsiella* spp., the accuracy of Carba-R was the highest (100.00%), followed by the CGI test (98.09%) and Carba 5 (96.82%). For *E. coli*, the accuracies of Carba 5 and the CGI test were both 100.00%, and that of Carba-R was 96.00%. Additionally, the accuracy of each assay varied with different types of carbapenemases, especially for the KPC variants and IMP variants.

Despite the fact that CZA was recently introduced for chemotherapy, the in vivo evolution of CZA-resistant strains after prolonged treatment were reported [14,15,16]. Previous studies demonstrated that the majority of CZA-resistant strains produced KPC-2 or KPC-3 variants [17,18,19]. The most frequently identified mutation was Asp^179^Tyr (D^179^Y), which was located within the omega loop of the KPC enzyme [17,18,19]. So far, 123 types of KPC variants have been deposited in the NCBI database, among which 50 types were resistant to beta-lactam/beta-lactamase inhibitor combinations (https://www.ncbi.nlm.nih.gov/pathogens/refgene/#KPC, accessed on 4 September 2022). In this study, 13 types of KPC variants were tested with the Carba 5, CGI test, and Xpert Carba-R, and KPC-2 and KPC-3 were accurately identified by all three methods. Although various studies have evaluated the performance of NG-test Carba 5 and Xpert Carba-R, KPC variants-producing strains were seldom studied. According to the previous studies [20,21], Carba 5 could detect KPC-3, KPC-14, KPC-35, KPC-78, and KPC-79. In the current study, Carba 5 could detect KPC-3, KPC-25, KPC-35, KPC-51, KPC-78, and KPC-93, while KPC-33, KPC-52, KPC-71, KPC-76, KPC-77, and KPC-123 were not detected by Carba 5. KPC-33, KPC-52, and KPC-76 all possessed the D^179^Y substitution, KPC-71 had an amino acid insertion at position 181, KPC-77 had an amino acid substitution at position 164, and KPC-123 had amino acid insertions at positions 179 and 270. The majority of those mutations were located inside the omega loop of KPC enzymes (R164-D179). To be concerned, some KPC variants such as KPC31 and KPC33 could no longer be considered as carbapenemases, as they had lost their carbapenemase activity. In this study, KPC-33, KPC-52, KPC-71, and KPC-123 belonged to cephalosporinases rather than carbapenemases, while KPC-76 and KPC-77 had poor carbapenemase activity. As opposed to Queslati’s study [22], which reported that NG-test Carba 5 demonstrated a sensitivity of 100.00% for the detection of KPC enzymes with carbapenemase activity, in the current study, NG-test Carba 5 missed two KPC-type carbapenemases with low activity. As for the CGI test, ten types of KPC variants were accurately identified, including KPC-3, KPC-25, KPC-33, KPC-35, KPC-51, KPC-52, KPC-71, KPC-77, KPC-78, and KPC-93, but two variants, KPC-76 and KPC-123, were missed. Distinct from the former two methods, Carba-R was based on real-time PCR, which was less affected by gene mutations. Carba-R demonstrated a perfect performance in the detection all of the KPC variants, with a sensitivity of 100.00% and a specificity of 100.00%, which was in line with the findings of Ding et al. [20].

Compared with KPC, IMP is less prevalent in China. According to our previous nationwide surveillance of clinical CRE strains [23], 3% CRE produced IMP carbapenemase, all of which was encoded by *bla*_IMP-4_. In this study, six variants of IMP were included, and NG-test Carba 5 successfully identified all of these IMP variants, including IMP-1, IMP-4, IMP-8, IMP-25, IMP-26, and IMP-30. As was reported in previous studies [24,25,26,27,28], NG-test Carba 5 was designed to cover almost all IMP variants, including IMP-1, IMP-2, IMP-4, IMP-5, IMP-6, IMP-7, IMP-8, IMP-10, IMP-11, IMP-19, IMP-26, IMP-29, IMP-39, and IMP-79. However, due to the diversity of the IMP variants, some false-negative results were also reported, such as for IMP-13, IMP-14, IMP-15, IMP-18, IMP-37, IMP-63, IMP-66, and IMP-71 [24,26,27]. In comparison, the CGI test accurately identified nine strains with IMP variants (including IMP-1, IMP-4, IMP-8, and IMP-25) and reported four false-negative results. Interestingly, a recent study by Zhang et al. [8] reported that the CGI test failed to detect IMP-8; however, in the current study, IMP-8 was successfully identified in *P. aeruginosa*, but there was a failure to detect IMP-8 in *A. hydrophila*. The distinct expression levels of IMP-8 in different strains might throw light upon the contradiction. Regarding Xpert Carba-R, the overall sensitivity was 76.92%, and Carba-R successfully detected IMP-1, IMP-25, IMP-26, and IMP-30. Confusingly, a *bla*_IMP-4_-carrying *A. hydrophila* was misidentified as NDM-producing, whilst a *bla*_IMP-4_-carrying *K. oxytoca* was accurately identified. Two IMP-8-producing strains were identified as false-negative by Carba-R. According to the manufacturer’s datasheet, Carba-R was designed to detect IMP-4 and IMP-8 based on in silico analysis; nevertheless, IMP-8 could not be detected in several reports [8,29]. The low copy number of the carbapenemase genes in the tested strains might be the reason for the failure in the detection by Carba-R. Additionally, various mutations located in primer- or probe-binding regions might affect the effective detection by Carba-R.

Regarding strains with multiple carbapenemase genes, NG-test Carba 5 successfully identified all these carbapenemases. In comparison, the CGI test missed IMP-4 in five strains, including three strains co-producing KPC-2 and IMP-4 and two strains co-producing NDM-1 and IMP-4. IMP-4 was perfectly detected in strains with a single carbapenemase gene, as shown in our study. Interferences caused by other antibodies might exist, which could affect the co-detection of multiple resistance genes. As for Xpert Carba-R, a missed detection of IMP-4 in one strain of *K. michiganensis* co-carrying *bla*_KPC-2_, *bla*_NDM-1_, and *bla*_IMP-4_ was reported. Additionally, only IMP was reported in two *P. aeruginosa* strains co-producing IMP-45 and NDM-1. The low copy number of the associated genes might be the reason for the false-negative results. Overall, for strains with multiple carbapenemase genes, other regular methods such as antimicrobial susceptibility testing and phenotypic characterization should be conducted to resolve the resistance mechanisms.

Our study has some limitations. First, only one VIM-producing strain and two OXA-48-like-producing strains were included here; thus, the performance of these methods in the detection and VIM and OXA-48-like carbapenemases should be further evaluated in future studies. Purified colonies were subjected to the detection in this study, which might not accurately reflect the performance of detection methods; thus, clinical samples should be included in further studies. 

In conclusion, we conducted a comparison of the performances of two lateral flow immunochromatographic assays, namely, NG-test Carba 5 and the CGI test, and the automatic real-time quantitative PCR Xpert Carba-R in carbapenemases detection. In comparison with the WGS results, all three methods demonstrated a decent accuracy and high accordance. However, the sensitivity and specificity of each method varied for different kinds of carbapenemases. For the detection of KPC variants, especially for those CZA-resistant strains, PCR-based Xpert Carba-R was superior to the other two methods. For the detection of IMP, Carba 5 was more potent than Carba-R and the CGI test. Notably, different methods including antimicrobial susceptibility testing should be considered for strains with negative results in carbapenemase detection.

## 4. Materials and Methods

### 4.1. Strain Source

A total of 207 carbapenemase-producing strains collected from patients and hospital sewages were enrolled in this study, including *Escherichia coli* (n = 36), *Klebsiella pneumoniae* (n = 125), *Klebsiella oxytoca* (n = 25), *Klebsiella variicola* (n = 1), *Klebsiella michiganensis* (n = 1), *Enterobacter kobei* (n = 1), *Enterobacter cloacae* (n = 2), *Enterobacter xiangfangensis* (n = 1), *Citrobacter freundii* (n = 3), *Citrobacter koseri* (n = 1), *Raoultella ornithinolytica* (n = 2), *Aeromonas hydrophila* (n = 3), and *P. aeruginosa* (n = 6). The strains were inoculated onto Columbia blood agar (bioMérieux, Marcy l’Etoile, France) and cultivated overnight at 35 °C with 5% CO_2_. Vitek MS (bioMérieux, Marcy l’Etoile, France) was used for the preliminary species identification, and whole-genome sequencing was performed for the accurate identification of species and resistance genes.

### 4.2. Whole-Genome Sequencing and Genome Analysis

The Hipure Bacterial DNA kit (Magen, Shanghai, China) was used for the DNA extraction. The extracted DNA was subjected to WGS sequencing by Illumina (Illumina, San Diego, CA, USA). The reads were de novo assembled by SPAdes v3.13.1, and the carriage of antimicrobial resistance genes was identified at the Center for Genomic Epidemiology (CGE) (http://www.genomicepidemiology.org/services/, accessed on 4 September 2022) using ResFinder 4.1.

### 4.3. NG-Test Carba 5 Assay

According to the manufacturer’s instructions, the NG-test Carba 5 assay (NG Biotech, Guipry, France) was used for the detection of carbapenemases including KPC, IMP, NDM, VIM, and OXA-48-like. A colony of a pure cultivated strain was mixed with five drops of lysis buffer. After vortexing, the mixture should be left at 20–25 for ten minutes. Then, 100 μL of the mixture was transferred to the NG-Test Carba5 cassette. The results were read after 15 min of incubation.

### 4.4. Colloidal Gold Immunoassay (CGI) Test

A CGI test (Gold Mountain River Tech Development Company, Beijing, China) was developed based on colloidal gold immunochromatography to identify five major types of carbapenemases, including KPC, IMP, NDM, VIM, and OXA-48-like. Briefly, ten drops of the treatment solution and one or two colonies were vortexed together. Then, 50 μL of the mixture was added into the sample well of the testing cassettes, and the results were read in 10 min.

### 4.5. Xpert Carba-R Assay

The automated real-time quantitative PCR-based GeneXpert Carba-R assay (Cepheid Inc., Sunnyvale, USA) was applied for the rapid detection of five major types of carbapenemases (including KPC, IMP, NDM, VIM, and OXA-48-like). As per the manufacturer’s instructions, 0.5 McFarland suspension was prepared with pure cultivated colonies, and 10 μL of the suspension was mixed with 5 ml of the sample reagent and vortexed for ten seconds. Then, 1.7 mL of the mixture was added into the Xpert Carba-R assay sample well and run with the GeneXpert system.

### 4.6. Statistical Analysis

The sensitivity and specificity of the NG-test Carba 5, CGI test, and Xpert Carba-R were calculated with a 95% confidence interval (CI). The Kappa index (κ index) was used for the evaluation of the agreement between each method and WGS, the gold standard. The data were analyzed using SPSS Statistics v20.0 (IBM, Chicago, IL, USA).

## Figures and Tables

**Table 1:** Comparison of the NG-test Carba 5, colloidal gold immunoassay (CGI) test, and Xpert Carba-R.

Genes	NG-Test Carba 5							CGI Test							Xpert Carba-R						
	TP	FP	FN	TN	Se (95% CI)	Sp (95% CI)	κ (95% CI)	TP	FP	FN	TN	Se (95% CI)	Sp (95% CI)	κ (95% CI)	TP	FP	FN	TN	Se (95% CI)	Sp (95% CI)	κ (95% CI)
*bla*_KPC_	127	0	6	59	95.49 (90.02–98.15)%	100.00 (92.38–100.00)%	0.93 (0.87–0.99)	131	0	2	59	98.50 (94.12–99.74)%	100.00 (92.38–100.00)%	0.98 (0.92–1.00)	133	0	0	59	100.00 (96.50–100.00)%	100.00 (92.38–100.00)%	1.00 (1.00–1.00)
*bla*_NDM_	44	0	0	148	100.00 (90.00–100.00)%	100.00 (96.85–100.00)%	1.00 (1.00–1.00)	44	0	0	148	100.00 (90.00–100.00)%	100.00 (96.85–100.00)%	1.00 (1.00–1.00)	43	1	1	147	97.73 (86.49–99.88)%	99.32 (95.73–99.96)%	0.97 (0.93–1.00)
*bla*_IMP_	13	0	0	179	100.00 (71.66–100.00)%	100.00 (97.38–100.00)%	1.00 (1.00–1.00)	9	0	4	179	69.23 (38.89–89.64)%	100.00 (97.38–100.00)%	0.81 (0.62–0.99)	10	0	3	179	76.92 (45.98–93.83)%	100.00 (97.38–100.00)%	0.86 (0.71–1.00)
*bla*_OXA-48-like_	2	0	0	190	100.00 (19.79–100.00)%	100.00 (97.53–100.00)%	1.00 (1.00–1.00)	2	0	0	190	100.00 (19.79–100.00)%	100.00 (97.53–100.00)%	1.00 (1.00–1.00)	2	0	0	190	100.00 (19.79–100.00)%	100.00 (97.53–100.00)%	1.00 (1.00–1.00)

TP, true-positive; FP, false-positive; FN, false-negative; TN, true-negative; Se, sensitivity; Sp, specificity.

**Table 2:** Detection results of KPC variants by different methods.

Cabapenemase Genes	Amino Acid Substitution	Susceptibility of CZA	Organism(n)	Detection Results		
				NG-Test Carba 5	CGI Test	Xpert Carba-R
*bla*_KPC-2_		S	*Klebsiella pneumoniae*(120)	KPC	KPC	KPC
*bla*_KPC-3_		S	*Klebsiella pneumoniae*(1)	KPC	KPC	KPC
*bla*_KPC-25_	ins166_EL	S	*Klebsiella pneumoniae*(1)	KPC	KPC	KPC
*bla*_KPC-33_	D179Y	R	*Klebsiella pneumoniae*(1)	-	KPC	KPC
*bla*_KPC-35_	L196P	R	*Klebsiella pneumoniae*(1)	KPC	KPC	KPC
*bla*_KPC-51_	D179N + Y241H + H274N	R	*Klebsiella pneumoniae*(1)	KPC	KPC	KPC
*bla*_KPC-52_	D179Y + ins262_V	R	*Klebsiella pneumoniae*(1)	-	KPC	KPC
*bla*_KPC-71_	ins182_S	R	*Klebsiella pneumoniae*(1)	-	KPC	KPC
*bla*_KPC-76_	D179Y + 262V_268N dup	R	*Klebsiella pneumoniae*(1)	-	-	KPC
*bla*_KPC-77_	R164P	S	*Klebsiella pneumoniae*(1)	-	KPC	KPC
*bla*_KPC-78_	D179A	R	*Klebsiella pneumoniae*(1)	KPC	KPC	KPC
*bla*_KPC-93_	ins267_PNNRA	R	*Klebsiella pneumoniae*(1)	KPC	KPC	KPC
*bla*_KPC-123_	ins179_TY + ins270_DDKHSEA	R	*Citrobacter koseri*(1)	-	-	KPC

R, resistant; S, susceptibility; -, negative.

**Table 3:** Detection results of IMP variants by different methods.

Cabapenemase Genes	Number	Organism(Number)	Detection Results		
			NG-Test Carba 5	CGI Test	Xpert Carba-R
*bla*_IMP-1_	2	*Pseudomonas aeruginosa*(2)	IMP	IMP	IMP
*bla*_IMP-4_	4	*Klebsiella oxytoca*(3)	IMP	IMP	IMP
		*Aeromonas hydrophila*(1)	IMP	IMP	NDM
*bla*_IMP-8_	2	*Pseudomonas aeruginosa*(1)	IMP	IMP	-
		*Aeromonas hydrophila*(1)	IMP	-	-
*bla*_IMP-25_	1	*Pseudomonas aeruginosa*(1)	IMP	IMP	IMP
*bla*_IMP-26_	2	*Klebsiella pneumoniae*(2)	IMP	IMP	IMP
			IMP	-	IMP
*bla*_IMP-30_	2	*Klebsiella pneumoniae*(2)	IMP	-	IMP
			IMP	-	IMP

**Table 4:** Detection results of multi-carbapenemase genes by different methods.

Carbapenemase Genes	Organism(n)	Detection Results		
		NG-Test Carba 5	CGI Test	Xpert Carba-R
*bla*_KPC-2_ + *bla*_IMP-4_	*Klebsiella pneumoniae*(1)	KPC + IMP	KPC	KPC + IMP
*bla*_KPC-2_ + *bla*_IMP-4_	*Klebsiella variicola* (1)	KPC + IMP	KPC	KPC + IMP
*bla*_KPC-2_ + *bla*_IMP-4_	*Klebsiella oxytoca*(1)	KPC + IMP	KPC	KPC + IMP
*bla*_KPC-2_ + *bla*_NDM-1_	*Citrobacter freundii*(1)	KPC + NDM	KPC + NDM	KPC + NDM
*bla*_KPC-2_ + *bla*_NDM-1_	*Citrobacter freundii*(1)	KPC + NDM	KPC + NDM	KPC + NDM
*bla*_KPC-2_ + *bla*_NDM-1_	*Klebsiella oxytoca*(1)	KPC + NDM	KPC + NDM	KPC + NDM
*bla*_KPC-2_ + *bla*_NDM-1_	*Enterobacter kobei*(1)	KPC + NDM	KPC + NDM	KPC + NDM
*bla*_KPC-2_ + *bla*_NDM-1_	*Klebsiella oxytoca*(1)	KPC + NDM	KPC + NDM	KPC + NDM
*bla*_KPC-2_ + *bla*_NDM-1_	*Raoultella ornithinolytica*(1)	KPC + NDM	KPC + NDM	KPC + NDM
*bla*_KPC-2_ + *bla*_NDM-1_ + *bla*_IMP-4_	*Klebsiella michiganensis* (1)	NDM + KPC + IMP	NDM + KPC + IMP	KPC + NDM
*bla*_IMP-45_ + *bla*_NDM-1_	*Pseudomonas aeruginosa*(1)	IMP + NDM	IMP + NDM	IMP
*bla*_IMP-45_ + *bla*_NDM-1_	*Pseudomonas aeruginosa*(1)	IMP + NDM	IMP + NDM	IMP
*bla*_NDM-1_ + *bla*_IMP-4_	*Klebsiella oxytoca*(1)	NDM + IMP	NDM	NDM + IMP
*bla*_NDM-1_ + *bla*_IMP-4_	*Klebsiella oxytoca*(1)	NDM + IMP	NDM	NDM + IMP
*bla*_VIM-1_ + *bla*_NDM-1_	*Enterobacter cloacae*(1)	VIM + NDM	VIM + NDM	VIM + NDM

## Data Availability

The data presented in this study are available in supplementary material.

## Supplementary Materials

The following supporting information can be downloaded at: https://www.mdpi.com/article/10.3390/antibiotics12020300/s1, Table S1: Detection results of carbapenemases genes by NG-test Carba 5, colloidal gold immunoassay (CGI) test, and Xpert Carba-R ; Table S2: Characteristics of strains tested in the current study.
